# Interaction between muscle tone, short-range stiffness and increased sensory feedback gains explains key kinematic features of the pendulum test in spastic cerebral palsy: A simulation study

**DOI:** 10.1371/journal.pone.0205763

**Published:** 2018-10-18

**Authors:** Friedl De Groote, Kyle P. Blum, Brian C. Horslen, Lena H. Ting

**Affiliations:** 1 Department of Movement Sciences, KU Leuven, Leuven, Belgium; 2 Wallace H. Coulter Department of Biomedical Engineering, Emory University and Georgia Institute of Technology, Atlanta, Georgia, United States of America; 3 Department of Rehabilitation Medicine, Division of Physical Therapy, Emory University, Atlanta, Georgia, United States of America; Fondazione Santa Lucia Istituto di Ricovero e Cura a Carattere Scientifico, ITALY

## Abstract

The pendulum test is a sensitive clinical assessment of spasticity where the lower leg is dropped from the horizontal position and features of limb motion are recorded. Three key kinematic features are associated with the degree of severity of spasticity in children with cerebral palsy: decreased initial limb excursion, reduced number of limb oscillations, and a non-vertical resting limb angle. While spasticity is attributed to increased velocity-dependent resistance to motion, prior models simulating increased sensorimotor feedback of muscle velocity fail to explain the key pendulum test kinematic outcomes in spastic individuals. Here we hypothesized that increased muscle tone, causing a transient increase in muscle force, i.e. short-range stiffness, could account for reduced first swing excursion and non-vertical resting limb angle. We further hypothesized that hyperreflexia modeled based on muscle fiber force, and not velocity, feedback would be necessary to reduce the number of oscillations because of its interaction with transiently increased muscle force due to short-range stiffness. We simulated the lower leg as a torque-driven single-link pendulum. Muscle tone was modeled as a constant baseline joint torque, short-range stiffness torque was dependent on the level of muscle tone, and delayed sensory feedback torque to simulate reflex activity was based on either muscle velocity or force. Muscle tone and transient short-range stiffness were necessary to simulate decreased initial swing excursion and non-vertical resting leg angle. Moreover, the reduction in the number of oscillations was best reproduced by simulating stretch reflex activity in terms of force, and not velocity, feedback. Varying only baseline muscle torque and reflex gain, we simulated a range of pendulum test kinematics observed across different levels of spasticity. Our model lends insight into physiological mechanisms of spasticity whose contributions can vary on an individual-specific basis, and potentially across different neurological disorders that manifest spasticity as a symptom.

## Introduction

The mechanisms of spasticity are poorly understood. Spasticity is a common impairment in cerebral palsy (CP) and stroke, and is traditionally defined as a velocity-dependent increase in tonic stretch reflexes resulting from hyperexcitability of the stretch reflex [1]. The Ashworth Scale or Modified Ashworth Scale are the principal methods for clinical assessment of spasticity. During these tests, the patient is asked to relax and an examiner rotates the joint under investigation at different speeds. Spasticity is subjectively evaluated as increased resistance to imposed joint motion as speed increases, which is accompanied by increased muscle contraction presumed to arise from exaggerated reflex responses. The limitations of such manual assessments are increasingly being acknowledged [2]. For example, the score depends on the examiner’s subjective interpretation of the resistance, the level of baseline muscle activity is not controlled, and the test does not distinguish between reflexive and non-reflexive components of the resistance, such as those resulting from increased baseline muscle activity and/or non-contractile tissue properties [2]. Henceforth, we will refer to baseline muscle activity as muscle tone. More quantitative tests of spasticity in combination with computational modeling to infer potential contributions of underlying neuromechanical mechanisms may be helpful to understand the mechanisms of spasticity, and ultimately how these mechanisms contribute to impaired sensorimotor behaviors.

**Fig 1:**
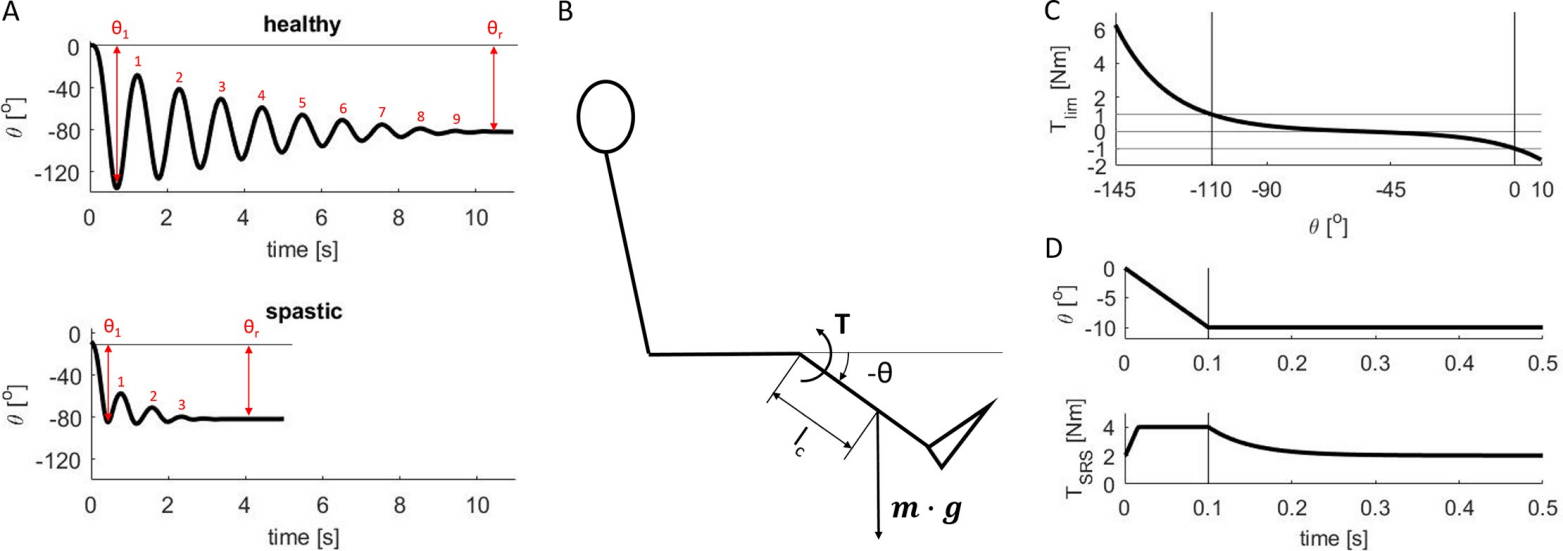
**A. Kinematic features of lower leg kinematics during the pendulum test.** Kinematics of the knee (joint angle θ) for a healthy individual and an individual with CP and moderate spasticity (reproduced from Fowler et al. [3]). First swing excursion θ_1_, number of oscillations, and resting angle θ_r_ are smaller for the individual with spastic CP than for the healthy individual. **B. Model of pendulum test.** A torque-driven pendulum represents the lower leg. The torque T resists the lower leg movement under the influence of gravity (product of lower leg mass m and gravitational acceleration g). **C. Coordinate limit torque.** Coordinate limit torque T_lim_, representing the increased joint stiffness towards the limits of the range of motion, as a function of knee joint angle. The interval of joint angles [θ_flex_ θ_ext_], for which passive stiffness is small, is indicated by vertical lines. **D. Short-range stiffness torque.** Short-range stiffness torque in response to a displacement of the knee joint for a baseline torque of 2Nm.

The pendulum test is an instrumented test that is more controlled than the Ashworth tests, and has been shown to be sensitive to the presence and severity of spasticity [3]. While the patient is seated, the examiner drops the lower leg from the horizontal position, with the knee joint extended; the lower leg is then allowed to swing freely under the influence of gravity and joint kinematics are recorded (Fig 1A and 1B). In healthy individuals, the swinging leg behaves as a damped pendulum, oscillating several times before coming to rest. As the severity of spasticity increases in children with CP (higher Modified Ashworth Score), three key changes in pendulum test kinematics have been found: 1) a decrease in the amplitude of the first swing excursion, 2) a decreased number of oscillations, and 3) a less vertical resting lower leg angle (Fig 1A)[3]. The pendulum test may also be more sensitive to symptoms of spasticity, as children with CP who lack signs of spasticity on a Modified Ashworth Scale also exhibit abnormal pendulum test kinematics. Overall, the decrease in the first swing excursion has been found to be the best predictor of spasticity severity, but the mechanisms underlying this key indicator of spasticity are poorly understood.

Previous neuromechanical models have had difficulty in reproducing key changes in pendulum test kinematics in spastic individuals, particularly the decreased first swing excursion [4,5,6,7]. Previous studies have focused on modeling the effects of increased reflex activity as the underlying cause of abnormal pendulum test kinematics. Reproducing the decreased amplitude of the first swing oscillation has been particularly difficult; to do so, prior models have required changing model parameter values in each oscillation or half-cycle, including sensorimotor feedback gains, thresholds, and/or reference positions (Table 1). The reduced number of oscillations has been simulated by altering reflex activation of muscles, either through increased sensory feedback gains or through reduced sensory feedback thresholds. Finally, only two models have reproduced a non-vertical resting leg angle, but only in the severest spasticity condition. However, experimental results show a progressive change in resting leg angle with severity of spasticity [3]. Moreover, simulating a non-vertical resting leg angle was previously achieved by imposing a non-vertical reference position on the length feedback loop, which has no clear physiological interpretation. Thus, while prior models can reproduce some of the features of leg swing kinematics, they require non-constant model parameters over the course of a single trial, as well as across symptom severity. As the models rely on variable parameters instead of explicitly describing hypothesized mechanisms of spasticity, the relationship of the varying model parameters to underlying physiological mechanisms is unclear.

**Table 1:** Overview of neuromechanical models of the pendulum test.

	Model	Mechanism explaining 1. reduced first swing excursion, 2. reduced number of oscillations, 3. non-vertical resting angle	Model parameters	Model parameters that are different in spastic and healthy subjects
Lin and Rymer, 1991	Torque-driven pendulum with time-varying stiffness and damping. Step changes in stiffness and damping describe spasticity. The spring has a variable resting angle.	1. Resting angle of passive spring. 2. Higher damping. 3. Not described by model. Non-vertical resting angles were considered measurement errors.	Stiffness K and damping B. Onset and end of spastic response τ_on_ and τ_off_. Stiffness and damping ‘gains’ k_K_ and k_B_. Spring resting angle θ_R._	K and B estimated per half cycle. τ_on_ = 30ms and τ_off_ = 140ms estimated from EMG. Gains estimated for first and second half cycle and scaled based on EMG for remainder of response. θ_R_ equals joint angle at EMG onset.
He et al., 1997	Pendulum actuated by five muscles. Spasticity described by non-linear feedback from muscle fiber length and velocity.	1. Low fiber length feedback threshold. 2. Reflex gains and thresholds. 3. Fiber length feedback with low reflex threshold.	Parameters of muscle-tendon dynamic model. Reflex delay τ = 30–60 ms. Four parameters describing length and velocity reflex thresholds and reflex gains. Task-dependent range of muscle stretch L_R_.	Reflex thresholds and gains of the five muscles were varied to describe different spastic responses. L_R_ varied as a function of the task (e.g. position of hip).
LeCavorzin et al., 2001	Torque-driven pendulum with linear stiffness and damping. Spasticity described by feedback from joint position and velocity.	1. Low/zero/negative velocity feedback gain in combination with low/zero/negative velocity threshold and constant position feedback gain. 2. Decrease in velocity feedback gain and threshold, and increased damping. 3. Not described by model.	Stiffness K and damping B. Reflex delay τ = 30ms. Variable *a* describing velocity threshold and velocity gain. Common position and velocity gain Γ. Scaling factor of velocity gain n. Angular variation during stretch δθ.	B was higher in spastic than in control subjects. *a* was varied to describe different spastic responses.
Fee and Foulds, 2004	Pendulum driven by extensor and flexor torque. Non-linear velocity feedback described spasticity.	1. Velocity feedback with delay of 120-140ms. 2. Velocity feedback. 3. Not described by model.	Extensor and flexor stiffness K and damping B. Varying reflex gains, onset times and delays.	Flexor and extensor contributions to spastic responses were each described by three reflex gains, onset times and delays.
**De Groote et al., current study**	Torque-driven pendulum with passive stiffness and damping and short-range stiffness. Spasticity described by constant torque representing muscle tone and feedback from joint position/velocity or active joint torque/derivative of torque.	1. Muscle short-range stiffness, muscle tone, and feedback from position/velocity or muscle torque/derivative of torque. 2. Feedback from position/velocity or torque/derivative of torque. 3. Muscle tone.	Stiffness k_lim_ and damping B. Short-range stiffness constant k_SRS_, critical stretch Δθ_c_, and decay time constant τ_SRS_. Baseline torque T_b_. Reflex delay *γ* = 50ms. Feedback gains k_x_ and k_dx_.	Higher baseline torque in children with CP. k_x_ and k_dx_ were varied to describe different spastic responses.

Muscle tone is often increased in individuals with spasticity, but this increased muscle tone has not been explicitly simulated in prior models. A broader definition describes spasticity as a “disordered sensory-motor control resulting from an upper motor neuron lesion, presenting as intermittent or sustained involuntary activation of muscles” [8]. None of the prior models of spasticity explicitly simulated muscle tone. One reason may be that the background muscle activity and the resulting joint stiffness are difficult to assess in an absolute sense. In prior models, any effects of increased muscle tone were likely lumped into parameters representing stiffness and damping of passive, i.e. non-contractile, tissue. We hypothesized that explicitly including muscle tone in simulations could better account for leg resting angle in a physiologically relevant manner.

Muscle short-range stiffness also contributes to muscle force during stretch and could contribute to the decreased first swing excursion. When muscle fibers are stretched after being held at a constant length for a period of time, short-range stiffness causes a rapid and transient increase in muscle force that depends on the level of isometric muscle activity and force prior to stretch [9,10]. The contribution of short-range stiffness has also been quantified in whole limb perturbations [11], and we recently showed that short-range stiffness plays a role in stabilizing posture against perturbations to standing balance [12]. When limb posture or standing balance is perturbed, isometrically activated muscles are rapidly stretched. We hypothesized that short-range stiffness also plays a role in the pendulum test where the leg is first held horizontally and then released such that the quadriceps are stretched by the force of gravity starting from an isometric condition. Moreover, short-range stiffness increases as the level of background muscle tone is increased due to the higher number of attached cross-bridges. Therefore, we hypothesized that the interaction between background muscle tone and short-range stiffness could be responsible for decreased first swing excursion in spasticity.

Muscle force due to short-range stiffness has further been shown to be encoded in muscle spindle sensory recordings, suggesting that reflex contributions to pendulum test kinematics could also be dependent on muscle force. Our recent work demonstrated that muscle short-range stiffness underlies history-dependent muscle spindle activity, in which sensory signals from muscle spindles are transiently increased in response to muscle stretch after being held at a constant length [13]. The increased muscle spindle response can be attributed to the encoding of a history-dependent increase in muscle force and rate change in force due to short-range stiffness. Since the magnitude of short-range stiffness is dependent on muscle activity, increased muscle tone would be predicted to increase both the muscle force due to short-range stiffness and the transient muscle spindle sensory signals driving stretch reflex responses. During conditions in which history-dependent muscle force due to short-range stiffness is observed, muscle spindle firing is more closely correlated to muscle fiber force and rate change in force than to muscle length and velocity. Therefore, we hypothesized that feedback from force-related variables in simulations could better account for changes in pendulum test kinematics with increasing levels of spasticity.

We tested the hypothesis that leg kinematics during the pendulum test in spastic individuals could be parsimoniously explained by increased muscle tone and sensory feedback gains if their interactions with muscle short-range stiffness are taken into consideration. We simulated the dynamics of a pendulum test based on a torque-driven biomechanical model of the lower leg (Fig 1B–1D). Our model consisted of a planar lower leg segment with passive stiffness and damping to simulate non-contractile musculotendon properties. Active joint torques representing muscle contractile behavior consisted of a constant baseline torque to represent increased muscle tone, a short-range stiffness torque dependent on the level of muscle tone, and a delayed sensory feedback torque to simulate reflex activity. Muscle short-range stiffness was scaled as a function of muscle tone. We simulated the reflex contributions to the pendulum test by modeling sensory feedback pathways based on either joint position and velocity to represent muscle length and velocity, or active torque and derivative of active torque to represent muscle fiber force and derivative of force. All model parameter values were held constant over the time course of each simulation. To simulate different degrees of spasticity, we altered both muscle tone and the sensitivity of the simulated reflex pathways, i.e. feedback gain values. Our evaluations of the model were based on published kinematic trajectories of the pendulum test in individuals with CP [3], but not electromyographic recordings of muscle activity. We were able to reproduce all three key features of the pendulum test associated with increased spasticity: 1) reduced amplitude of the first swing excursion, 2) reduced number of oscillations, and 3) less vertical resting angle [3]. Our results suggests that kinematic features of the pendulum test in individuals with spasticity due to CP result from an interaction between muscle tone, short-range stiffness, and force-related, but not velocity-related, reflex activity.

## Results

### Example simulations of healthy and spastic pendulum test kinematics

**Fig 2:**
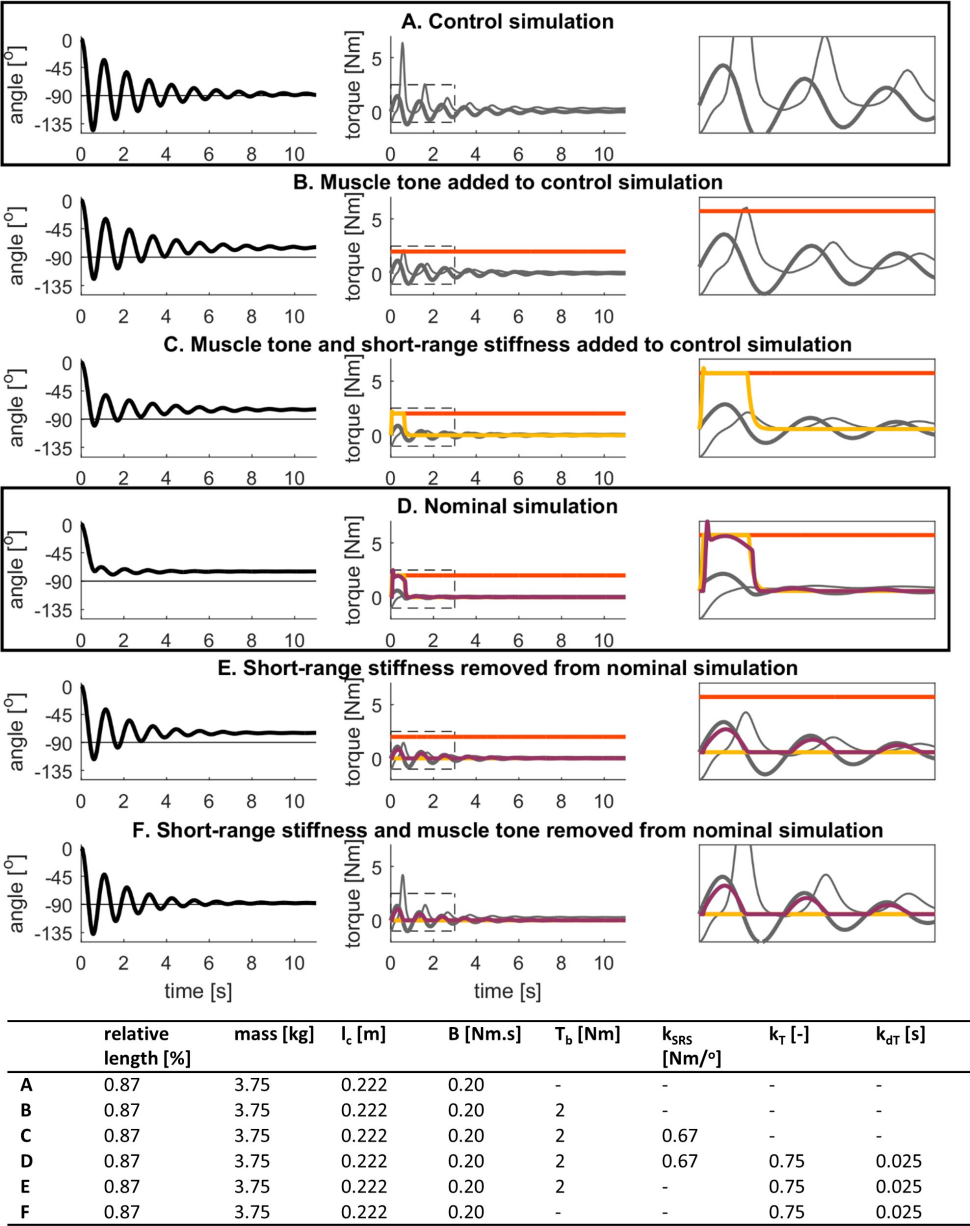
Example simulated lower-limb swing kinematics and torques during the pendulum test. Model parameters are reported in the table. Relative length is with respect to the length of the generic model [14], mass is the mass of the lower leg, and l_c_ is the distance between the knee joint center and the center of mass of the lower leg. **(A)** Lower leg segment actuated with a passive torque consisting of a damping torque, T_d_ (damping constant B); and a coordinate limit torque, T_lim_. **(B)** Lower leg segment actuated by a passive torque and a baseline torque representing muscle tone, T_b_ = 2Nm. **(C)** Lower leg segment actuated by a passive torque and a baseline torque when muscle short-range stiffness is accounted for by T_SRS_ (short-range stiffness constant k_SRS_). **(D)** Lower leg segment actuated by a passive torque, a baseline torque, a short-range stiffness torque and a delayed sensory feedback torque to simulate reflex activity, T_R_ (torque and derivative of torque reflex gains k_T_ and k_dT_). **(E)** Lower leg segment actuated by a passive torque, a baseline torque, and a delayed sensory feedback torque. **(F)** Lower leg segment actuated by a passive torque and a delayed sensory feedback torque.

We generated a control pendulum test simulation that reproduced kinematics of healthy individuals using only passive elements, that is without simulating baseline muscle tone nor reflex activity. Using only passive torque (damper and coordinate limit torque), we reproduced kinematic features of lower leg kinematics (Fig 2A) where the first swing excursion of 145.5^o^, 9 oscillations, and near-vertical resting angle of 88^o^ were all within one standard deviation of measured values for healthy individuals (Table 2, [3]). The coordinate limit torque T_lim_ limited leg flexion and mainly contributed at the end of the first and second flexion excursions whereas the passive damping torque T_d_ induced the damped behavior (Fig 2A, middle and right column). In the absence of baseline torque, there was no contribution of short-range stiffness torque (Eq 6).

**Table 2:** Key kinematic features of the pendulum test in spasticity as reported by Fowler et al. (2000). Individuals with CP were divided in three groups based on the Modified Ashworth Score (MAS) of the quadriceps.

	MAS	First swing excursion	Number of oscillations	Resting angle
Healthy	1	136.2^o^ ± 12.4^o^	8.0 ± 1.1	84.7^o^ ± 4.1^o^
CP–no spasticity	1	101.4^o^ ± 21.0^o^	5.6 ± 1.5	81.9^o^ ± 5.2^o^
CP–mild spasticity	2 or 3	77.2^o^ ± 23.8^o^	4.2 ± 1.3	73.6^o^ ± 9.3^o^
CP–severe spasticity	>3	44.1^o^ ± 12.2^o^	4.0 ± 1.2	69.5^o^ ± 11.1^o^

We generated a nominal pendulum test simulation of a spastic individual that reproduced all key changes in kinematic features of the pendulum test by introducing muscle tone, short-range stiffness, and non-zero reflex gains (Fig 2D). This nominal simulation of spasticity had a reduced first swing excursion of 74.5^o^, 3 oscillations, and a non-vertical resting angle of 74.8^o^, all within one standard deviation of measured values for CP idividuals with mild spasticity of the quadriceps (MAS of 2 or 3) (Table 2, [3]). In this nominal simulation of a spastic individual, baseline torque, short-range stiffness torque, and reflex torque all contributed similarly to the joint torque during the first swing thereby limiting the amplitude of the first swing excursion and the number of oscillations (Fig 2D, middle and right column). Whereas contributions from short-range stiffness and reflexes were small after the first swing, baseline torque counteracted the effect of gravity throughout the simulation and caused the non-vertical resting angle.

### Role of muscle tone, short-range stiffness, and stretch reflex gains in a nominal simulation of pendulum test kinematics in spasticity

To further understand how each mechanism in the nominal spastic simulation contributed to the key features of the pendulum test, we sequentially tested the contribution of each model component to the altered lower-limb swing kinematics (Fig 2A–2F). Starting from the control simulation of healthy pendulum kinematics (Fig 2A), we added the effects of muscle tone (Fig 2B), short-range stiffness (Fig 2C), and stretch reflexes (Fig 2D). From this nominal spastic model, we then removed the effects of short-range stiffness (Fig 2E), and muscle tone (Fig 2F). Results from this systematic investigation of the model behavior are reported below.

The primary effect of adding muscle tone in the absence of short-range stiffness was a less vertical resting angle, similar to that previously reported in individuals with CP (Fig 2B). In this simulation the baseline torque of 2Nm (less than 2% of maximal knee extension torque) counteracted the effect of gravity and caused a non-vertical resting angle of 74.8^o^, falling within one standard deviation of measured values for CP individuals with mild spasticity of the quadriceps (MAS of 2 or 3) (Table 2, [3]). First swing excursion and number of oscillations were also decreased (124.5^o^ and 8 oscillations), but were still within one standard deviation of measured values for healthy individuals.

When we included short-range stiffness that was proportional to muscle tone, the first swing excursion and number of oscillations decreased, resembling CP individuals *without* quadriceps spasticity (Fig 2C). The first swing excursion of 100.3^o^ and 7 oscillations were within one standard deviation of measured values for CP individuals without spasticity of the quadriceps (MAS of 1) (Table 2, [3]). The contributions of baseline torque and short-range stiffness torque to the total torque during the first swing excursion were similar in magnitude, so that both contributed about equally to reducing first swing excursion amplitude (Fig 2C, middle and right column). Adding short-range stiffness did not affect final resting angle.

Adding reflex activity further reduced first swing excursion and number of oscillations, to levels reported in CP individuals *with* quadriceps spasticity (Fig 2D, nominal simulation). We selected reflex gains that resulted in a first swing excursion amplitude that was smaller than the second swing excursion, as observed in spastic individuals. The first swing excursion of 74.5^o^, 3 oscillations, and resting angle of 74.8^o^ in our nominal simulation were all within one standard deviation of measured values for CP individuals with mild spasticity of the quadriceps (MAS of 2 or 3) (Table 2, [3]). Reflex torque contributed mainly during the first swing thereby reducing the first swing excursion and number of oscillations (Fig 2D, middle and right column).

Without short-range stiffness, we could not reproduce kinematics of an individual with quadriceps spasticity. The nominal simulation without short-range stiffness produced larger first swing excursion and more oscillations than would be anticipated for individuals with spastic quadriceps (Fig 2E). Despite the presence of reflex activity, the first swing excursion of 117.1^o^ and 6 oscillations were more than one standard deviation higher than measured values for CP individuals with spasticity of the quadriceps.

When both baseline tone and short-range stiffness were omitted in the presence of reflex activity, the first swing excursion of 137.5^o^ and baseline angle of 88^o^ resembled that of a healthy individual. However, the total of 6 oscillations was fewer than measured values for healthy individuals (Fig 2F).

### Interactions between baseline muscle tone and SRS on pendulum test kinematics

**Fig 3:**
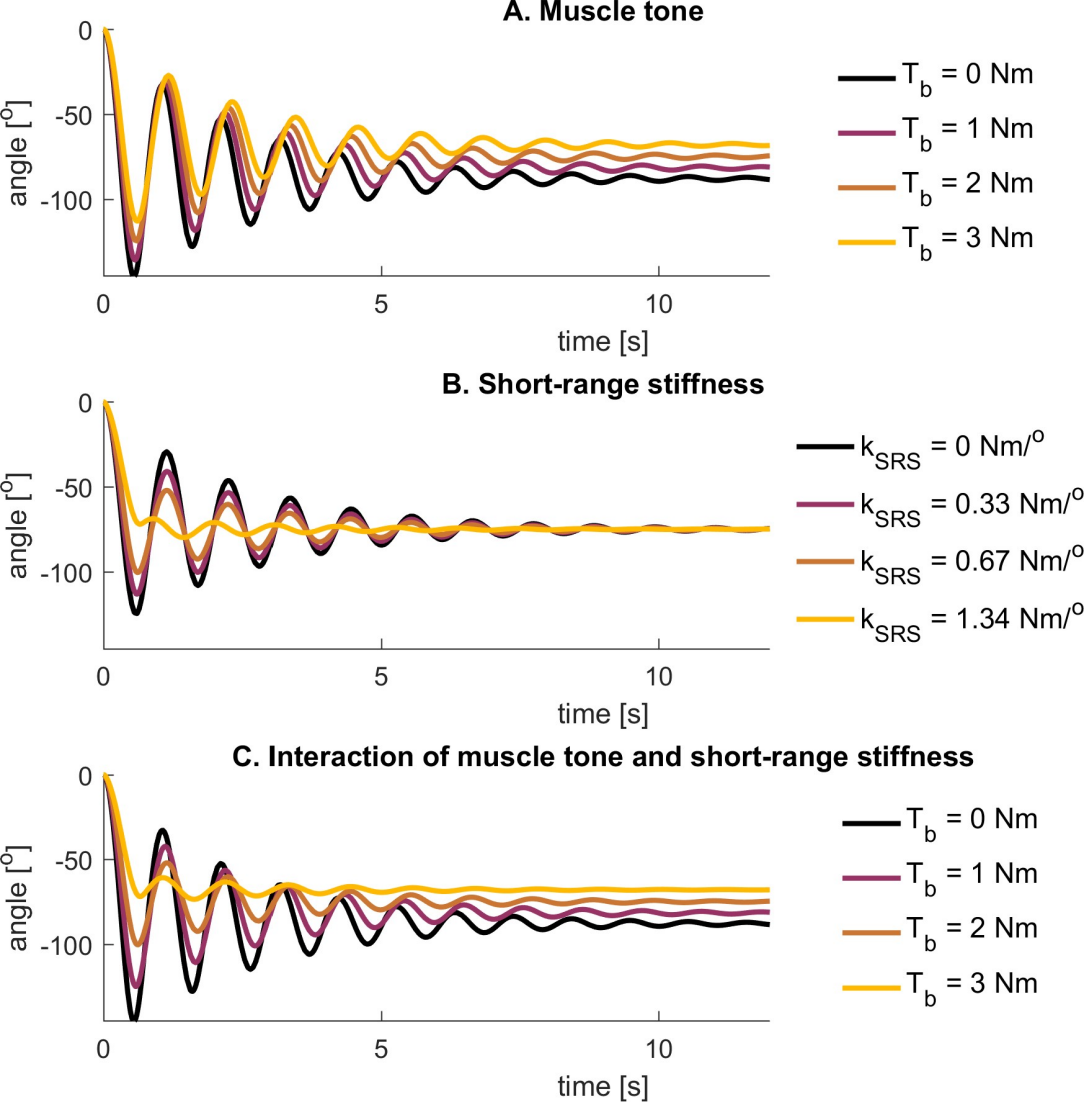
Simulated lower-limb swing kinematics during the pendulum test for different levels of baseline tone and short-range stiffness. **(A)** Simulated kinematics using a model with baseline tone T_b_ but without short-range stiffness. **(B)** Simulated kinematics using a model with baseline tone and short-range stiffness with different short-range stiffness constants k_SRS_. The baseline tone was constant (2Nm). **(C)** Simulated kinematics using a model with baseline tone and short-range stiffness. The short-range stiffness constant was 0.67Nm/deg.

Systematic variations of baseline muscle tone and short-range stiffness in the absence of reflex activity demonstrate the contributions of muscle tone to all three key features of pendulum test kinematics observed in individuals with CP. In the absence of short-range stiffness, varying muscle tone primarily affected resting angle (Fig 3A). In contrast, varying the short-range stiffness constant k_SRS_ while keeping tone constant affected the amplitude of the first swing excursion and the number of oscillations (Fig 3B). Systematic increases in baseline tone in the presence of a nominal level of short-range stiffness revealed interactions that caused the amplitude of the first swing excursion and the number of oscillations to decrease (Fig 3C). Because short-range stiffness is modulated by the amplitude of muscle tone, the short-range stiffness torque generated at the onset of the test was higher, reducing the first swing excursion, as well as the total number of oscillations.

### Effects of force-based and velocity-based reflexes on pendulum test kinematics

**Fig 4:**
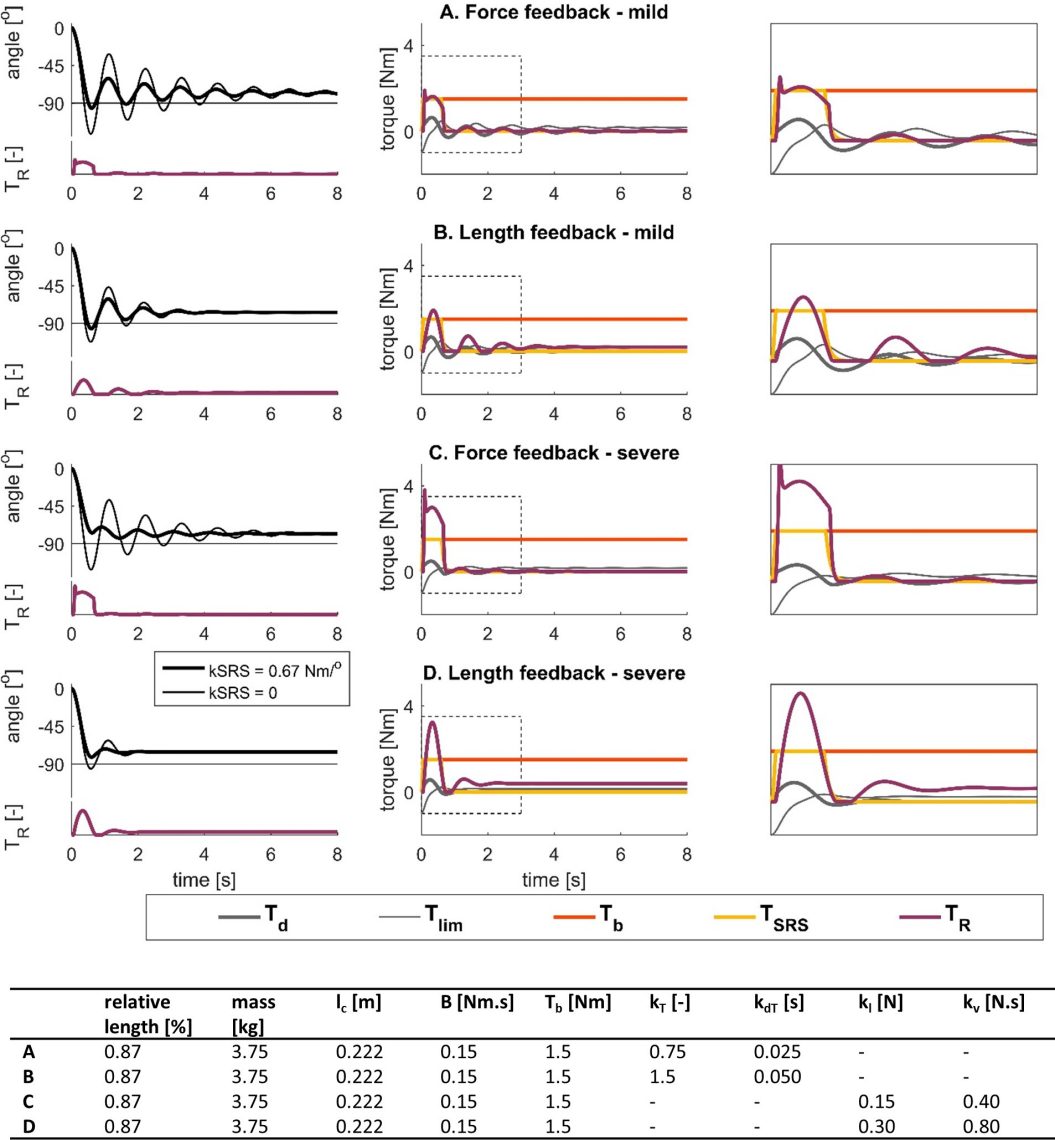
Simulated lower-limb swing kinematics and torque contributions during the pendulum test with different reflex models and reflex gains. Reflex torques scaled to mass and length of the individual are shown below the kinematic trajectories. Model parameters are reported in the table. Relative length is with respect to the length of the generic model [14]. **(A-B)** Simulated kinematics (left) and torque contributions for the simulations with short-range stiffness (middle, right) using a model with feedback from force and derivative of force. Feedback gains, k_T_ and k_dT_, were twice as large in B as in A. **(C-D)** Simulated kinematics (left) and torque contributions for the simulations with short-range stiffness (middle, right) using a model with feedback from length and velocity. Feedback gains, k_l_ and k_v_ were twice as large in D as in C. Reflex torques are shown below the kinematic trajectories.

To investigate the effects of force-based versus velocity-based feedback on pendulum test kinematics, we performed simulations with both reflex models where we kept all parameters constant and varied the reflex gains (Fig 4). Here we report example simulations of mild and severe spasticity using force-based feedback (Fig 4A and 4C) and using velocity-based feedback (Fig 4B and 4D). Velocity and force feedback models were matched to have similar first swing amplitudes and reflex torque amplitudes for the mild (Fig 4A and 4B) and severe cases (Fig 4C and 4D).

Our simulations of mild spasticity with the force-based and velocity-based models had similar first swing excursions (96.0^o^ and 96.4^o^, respectively) and resting angles (78.2^o^ and 76.9^o^, respectively) but the number of oscillations was higher for the force-based than for the velocity-based model (6 and 3 oscillations, respectively) (Fig 4A and 4B). Key kinematic features of both trajectories were within the range for individuals with CP with no (force-based model) or mild (velocity-based model) spasticity [3].

Doubling feedback gains in the force-based and velocity-based models reduced the amplitude of the first swing excursion (Fig 4C anf 4D) but only the force-based model resulted in a first swing excursion that was smaller than the second swing excursion and the resting angle, similar to observations in severely spastic individuals [3](Fig 4C). We were unable to reproduce this key feature with the velocity-based model (Fig 4D). In addition, doubling feedback gains reduced the number of oscillations in both models. However, the number of oscillations decreased below values reported for severely spastic individuals in the velocity-based model (Fig 4D). Increasing feedback gains had no effect on resting angle in the force-based model but resulted in a less vertical resting angle in the velocity-based model.

The interaction between force-based reflexes and muscle short-range stiffness was essential to reproduce kinematic trajectories of CP individuals with spastic quadriceps (Fig 4, thin lines). Short-range stiffness was required to reproduce a first swing excursion that was smaller than the resting angle. In addition, kinematic trajectories of CP individuals with spastic quadriceps reported by Fowler et al. [3] are characterized by either similar knee flexion angles at the end of the first and second excursion, or larger knee flexion at the end of the second excursion than at the end of the first excursion. We could only reproduce this feature when accounting for muscle short-range stiffness in the force-based model (Fig 4).

Characteristics of simulated reflex contributions were different in the force- and velocity-based models. In the force-based model, reflex torques increased sharply at the onset of the motion and had a nearly constant contribution during the first swing excursion where short-range stiffness torque was high, causing the amplitude of the first swing excursion and the number of oscillations to decrease without affecting the resting angle (Fig 4A and 4C). In the velocity-based reflex model, the reflex torque was also high during the first swing excursion but the rise and decay were slower. The high reflex torque ‘burst’ during the first swing was followed by smaller reflex torque ‘bursts’ during consecutive flexion excursions, causing the amplitude of the first swing excursion and the number of oscillations to decrease (Fig 4B and 4D). In addition, the length-related component of the reflex torque caused a less vertical resting angle.

### Interaction between baseline torque and reflex gains

**Fig 5:**
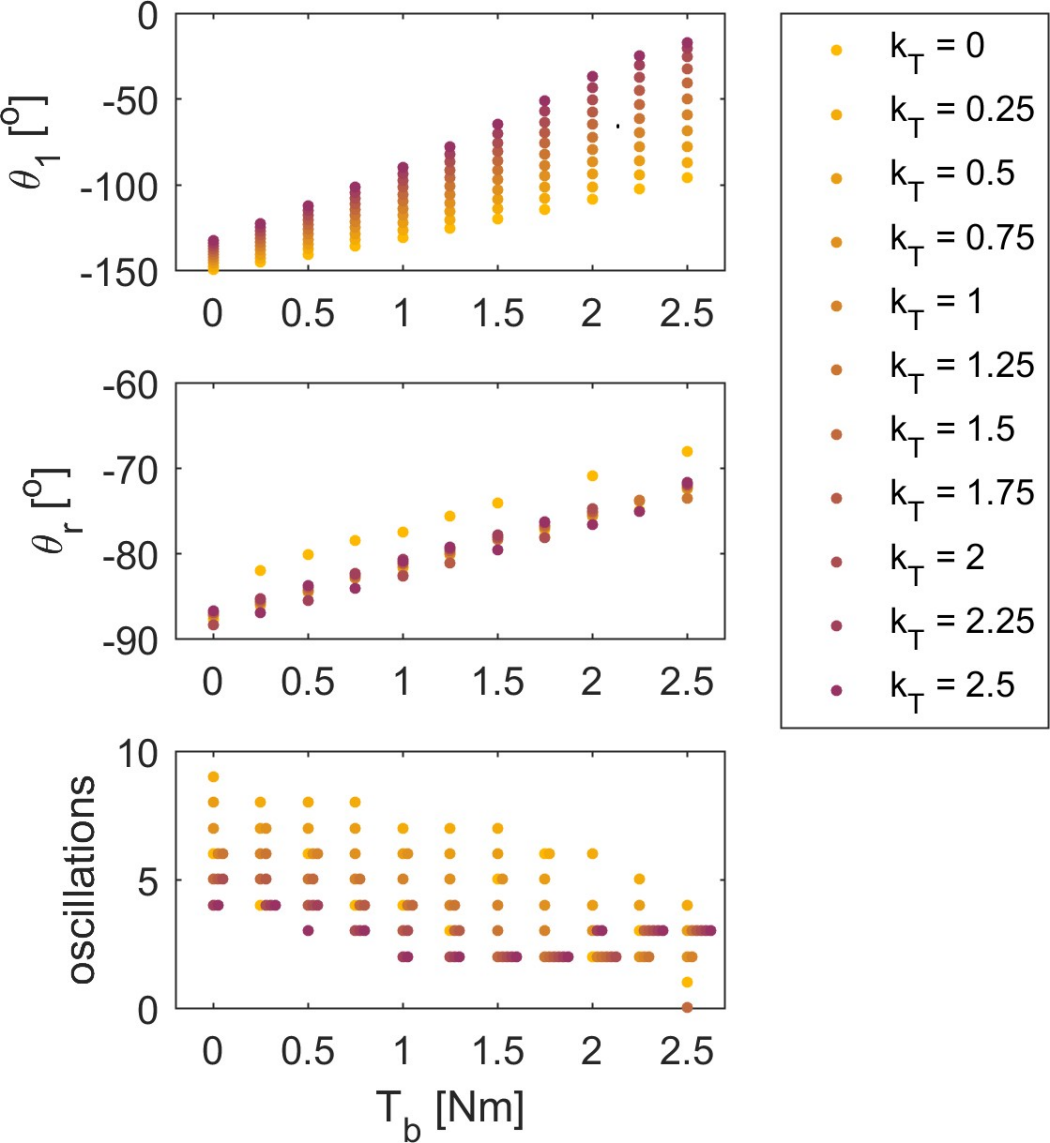
Key kinematic features of the pendulum test in spasticity as a function of baseline torque and feedback gain. Simulations were performed for combinations of 11 baseline torques and 11 torque feedback gains while keeping all other parameters constant. First swing excursion θ_1_ (top), resting angle θ_r_ (middle), and number of oscillations (bottom) are shown as a function of baseline torque T_b_. Values are color-coded according to torque feedback gain k_T_. Note that we represented simulations resulting in the same number of oscillations at a given baseline torque with horizontally spaced dots.

We systematically varied muscle tone and torque reflex gain (constant gain for derivative of torque) in the force-based model. First swing excursion decreased with both increasing baseline torque representing muscle tone and increasing torque reflex gain, but the effect of an increase in torque reflex gain was larger for larger baseline torques (Fig 5A). The coupling between muscle tone and reflex gains was due to muscle short-range stiffness being proportional to muscle tone (Fig 5B). The interaction between delayed sensory feedback and short-range stiffness due to increased muscle tone can explain the high sensitivity of first swing excursion to the level of spasticity (Table 2). The resting angle is less vertical as muscle tone increases but is not sensitive to torque feedback gain. The number of oscillations decreased both with increasing muscle tone and increasing torque reflex gain (Fig 5C).

### Reproduction of measured pendulum test kinematics

**Fig 6:**
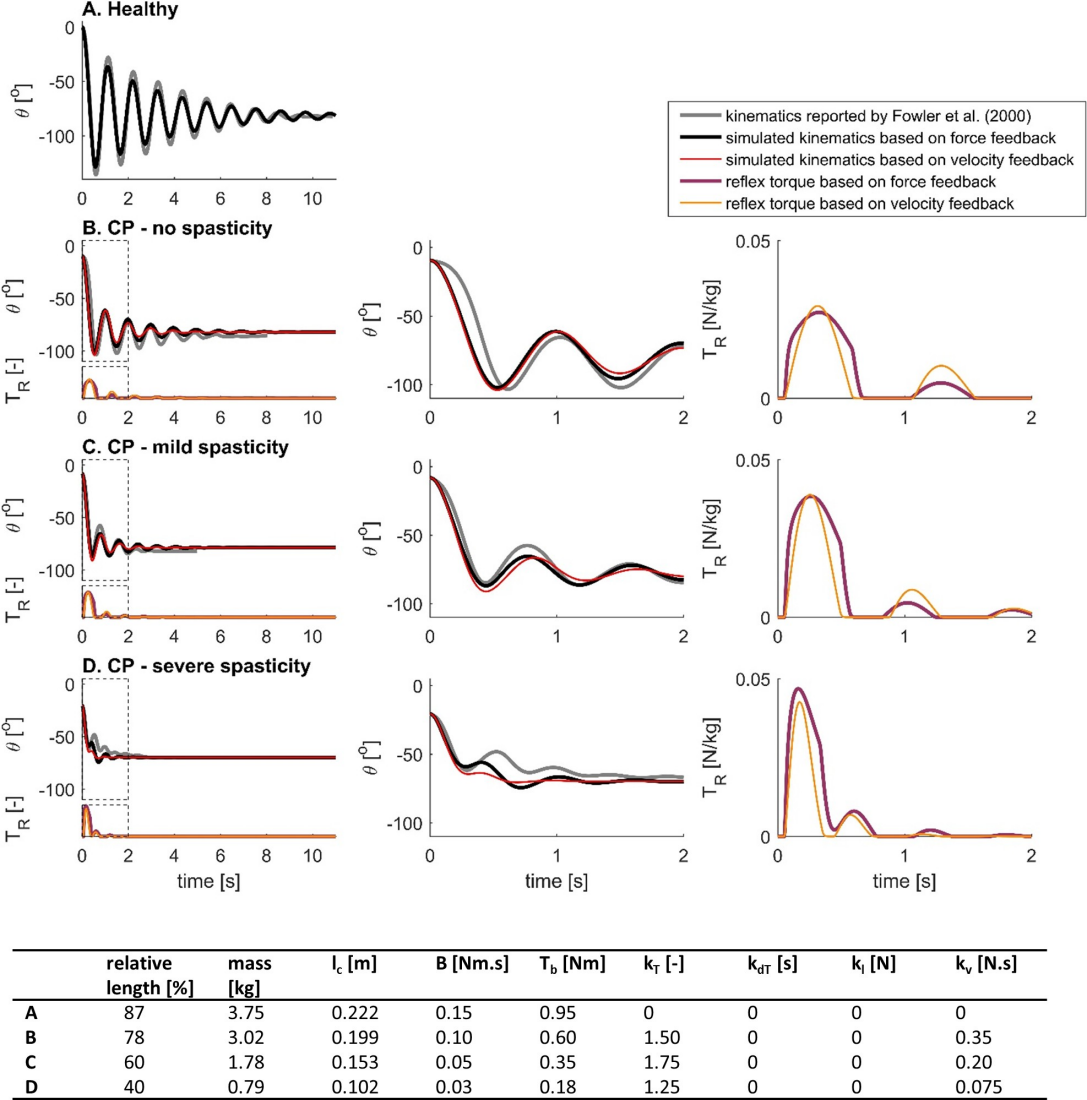
Comparison of simulated and experimental lower-limb swing kinematics for four individuals with different levels of spasticity. Experimental data from Fowler et al. ([3], Fig 3). The model parameters were manually tuned to obtain a good fit with the experimental data and are reported in the table. Relative length is with respect to the length of the generic model [14]. Swing kinematics were simulated using reflex torques based on feedback from force and derivative of force (black), and using reflex torques based on feedback from length and velocity (red). **(Left)** Reflex torques are shown below the kinematic trajectories. **(Middle)** Kinematics during the first 2s after release of the leg. **(Right)** Reflex torques scaled to mass and length of the individual during the first 2s after release of the leg.

The whole range of pendulum test kinematics observed in healthy individuals and CP individuals with different levels of spasticity could be simulated by varying muscle tone and sensory feedback gains in our model. We manually tuned baseline torque and reflex gains in both reflex models, as well as parameters related to subject size, to track previously published data from a healthy individual and three CP individuals with different levels of spasticity [3]. Since subjects in Fowler’s study were 7 to 50 years old and individual subjects’ length and mass were not reported, we chose the length such that simulated and measured frequencies of the oscillations in knee kinematics matched. We scaled lower leg length by relative length, i.e. the ratio between subject’s length and generic model’s length, and mass by relative length squared (see Eq 2). In addition, we reduced damping constant B with subject size. We obtained good fits between our simulations and published data from a healthy subject and three children with CP using both reflex models, although the force-based model reproduced kinematics of the severe spastic child slightly better than the velocity-based model (Fig 6).

We simulated healthy pendulum test kinematics using a small baseline torque and zero reflex gains (Fig 6A, table in Fig 6 for parameter values).

Three CP individuals with different levels of spasticity were simulated by tuning baseline torque and reflex gains in either the force-based (Fig 6B–6D, black lines) or velocity-based (Fig 6B–6D, red lines) reflex models. In the force-based reflex model, torque feedback gains were large and derivative of torque reflex gains were zero in all CP individuals (table in Fig 6). In the velocity-based reflex model, length feedback gains were zero and velocity reflex gains were large in all CP individuals (table in Fig 6).

Tracking results were slightly worse for the CP individual with severe spasticity (Fig 6D). Although we were able to reproduce the reduced amplitude of the first swing excursion and the resting angle, we were unable to track the knee joint angle accurately during the first and second oscillations (Fig 6D, middle). In addition, we were unable to reproduce the oscillatory behavior with the velocity-based model.

Reflex torques, normalized by subject mass and height, increased with the subject’s level of spasticity (Fig 6, right). In all simulations of spastic pendulum test kinematics, reflex activity was large during the first excursion and was small during consecutive flexion excursions (Fig 6, right column), which is in agreement with electromyography (EMG) signals reported for vastus lateralis [3].

## Discussion

Here we demonstrate a physiologically-plausible neuromechanical model that can parsimoniously reproduce key features of the pendulum test for spasticity across a range of severity levels. Despite the simplicity of our model, it revealed key physiological variables that could account for the spectrum of pendulum test behaviors seen across spasticity severity levels in individuals with CP. Our simulations reveal the critical role of increased muscle tone and muscle short-range stiffness rather than velocity-dependent feedback in reproducing three key kinematic features of the pendulum test associated with spasticity in individuals with CP [3]. In particular, baseline muscle tone was necessary to reproduce non-vertical resting leg angle. Further, transient short-range stiffness and its interaction with increased force-based reflexes were necessary to simulate the decreased initial swing excursion. These two key features of spasticity have previously been challenging to simulate. Notably, while spasticity is typically described as an increase in velocity-dependent reflex feedback, we found that the reduction in the number of oscillations was best reproduced by simulating stretch reflex activity in terms of force feedback. As such, our model suggests that increased muscle tone, history-dependent features of muscle short-range stiffness, and sensorimotor feedback are all dysregulated in spasticity in individuals with CP, with their interactions resulting in abnormal limb dynamics. In contrast to prior simulations of the pendulum test of spasticity, the parameters in our neuromechanical model are physiologically interpretable, and could be useful in identifying physiological mechanisms underlying experimentally-measured pendulum test outcomes on an individual-specific basis, potentially across different movement disorders in which spasticity manifests as a symptom.

Our simulations suggest that non-vertical resting leg angle that increases with the severity of spasticity in individuals with CP is due to increased muscle tone. Most previously proposed models of the pendulum test were not able to account for the non-vertical resting angle whereas He et al. described it using increased reflex muscle activity due to velocity feedback [5]. We also found that increased length feedback gains could result in a less vertical resting angle, but gain changes were not necessary when muscle tone was sufficiently high (Fig 6, baseline torque normalized to mass and length increases with spasticity severity). As far as we know, there is no evidence for increased muscle activation or sustained muscle spindle firing when the leg is at rest at the end of the pendulum test as compared to the initial condition. However, both sustained muscle activity after (e.g. [15,16]) and continuous background muscle activity during (e.g. [17]) imposed joint rotations as applied during clinical spasticity assessment have been reported in some cases. The contribution of increased muscle tone to altered pendulum kinematics in our simulations is also consistent with the high prevalence of dystonia, which is characterized by involuntary intermittent and sustained muscle contractions, together with spasticity in CP [18]. Further, spasticity in spinal cord injury has been shown to result from persistent involuntary activation of muscles [19].

Regardless of the type of reflex feedback in our model, short-range stiffness that increased proportionally to muscle tone was necessary in all of our simulations to reduce first swing excursion. Despite the fact that first swing excursion is the outcome measure that is most highly associated with degree of spasticity, prior models have been unable to account for this key feature. Our simulations thus suggest that increasing muscle tone with the severity of spasticity causes higher short-range stiffness that reduces the amplitude of leg excursion on the first swing [3]. Isolated muscle fiber studies have shown an activation-dependent and rapid increase in tension during stretch due to short-range stiffness if the fiber starts in the isometric condition, and this increase in tension is nearly absent when a second stretch cycle immediately follows the first [20]. Similarly, history-dependent joint stiffness that could be attributed to muscle mechanical properties has been observed at the onset of imposed joint rotations in the human metacarpal phalangeal [21] and ankle [22] joints, and with the stiffness increased with baseline joint torque level [21]. Short-range stiffness due to holding the limb isometric before dropping it during the pendulum test likely acts only during the first and not subsequent swing excursions. In our simple model of muscle short-range stiffness, the increase in short-range stiffness force was proportional to muscle tone, reflecting the activation-dependence of short-range stiffness. However, short-range has been observed in electrically quiescent muscle preparations (see e.g. [13]) and in the ankle joint of relaxed humans [23] suggesting that muscle short-range stiffness can occur in the absence of measureable electrical muscle activity. In our simplified model, short-range stiffness increased until a critical stretch was reached and was then kept constant until the end of the stretch. However, experiments in single muscles fibers exhibit a small decrease in force before the onset of the force plateau [20]. In our simulations, contributions of short-range stiffness were limited to first stretch cycle, but it is possible that short-range stiffness could contribute to a smaller extent in the consecutive stretches. A more detailed model of muscle short-range stiffness might better reproduce the bursts of reflex activity and pendulum kinematics during consecutive stretches in severe spasticity but would not alter the important role of short-range stiffness to the decreased first swing excursion. As such, our results demonstrate the need for muscle models that have a short-range stiffness component, and further studies examining the degree to which short-range stiffness varies as function of baseline muscle activity.

Trajectories of the pendulum test in individuals with severe spastic CP required interactions between force-based reflex feedback and short-range stiffness, and was not adequately simulated based on velocity-based reflex feedback. In mild spasticity, the effects of force-based and velocity-based reflexes could not be distinguished based on their kinematic trajectories. However, the velocity-based reflex model could not account for the most severe characteristics of spasticity: a first swing excursion that was smaller than the resting angle while still having multiple oscillations (see Table 2, CP–severe spasticity and Fig 6D). In contrast, the force-based model could reproduce the reduction in first swing excursion, but only in the presence of short-range stiffness. Using force-based reflexes is supported by our recent work showing that muscle spindle firing rates reflect muscle fiber force and its first time derivative, which causes a very high, transient, and history-dependent initial burst in feline muscle spindle afferents due to muscle short-range stiffness [13]. Similarly in a human, Day et al. reported a high tibialis anterior muscle spindle firing rate at the onset of oscillatory passive rotations of the ankle that was absent during consecutive oscillations (see Fig 4 in [24]). Further, Lin and Rymer reported higher reflex activity in the flexor pollicis longus during the first cycle of an oscillatory movement compared to subsequent oscillations that are kinematically identical (see Fig 6 in [25]). We therefore hypothesize that holding the muscle isometric for a period of time before releasing the leg at the beginning of the pendulum test causes high short-range stiffness at muscle stretch onset. In turn, this short-range stiffness causes a large initial burst in the muscle spindle feedback at the onset of stretch, which increases the reflex torque in the first oscillation compared to subsequent leg oscillations. Thus, the force-related feedback is a physiologically plausible mechanism that accounts for the decreased first swing excursion without dampening subsequent oscillations. However, our evaluation of the reflex feedback models was limited by the lack of available muscle activity (electromyography) recordings.

**Fig 7:**
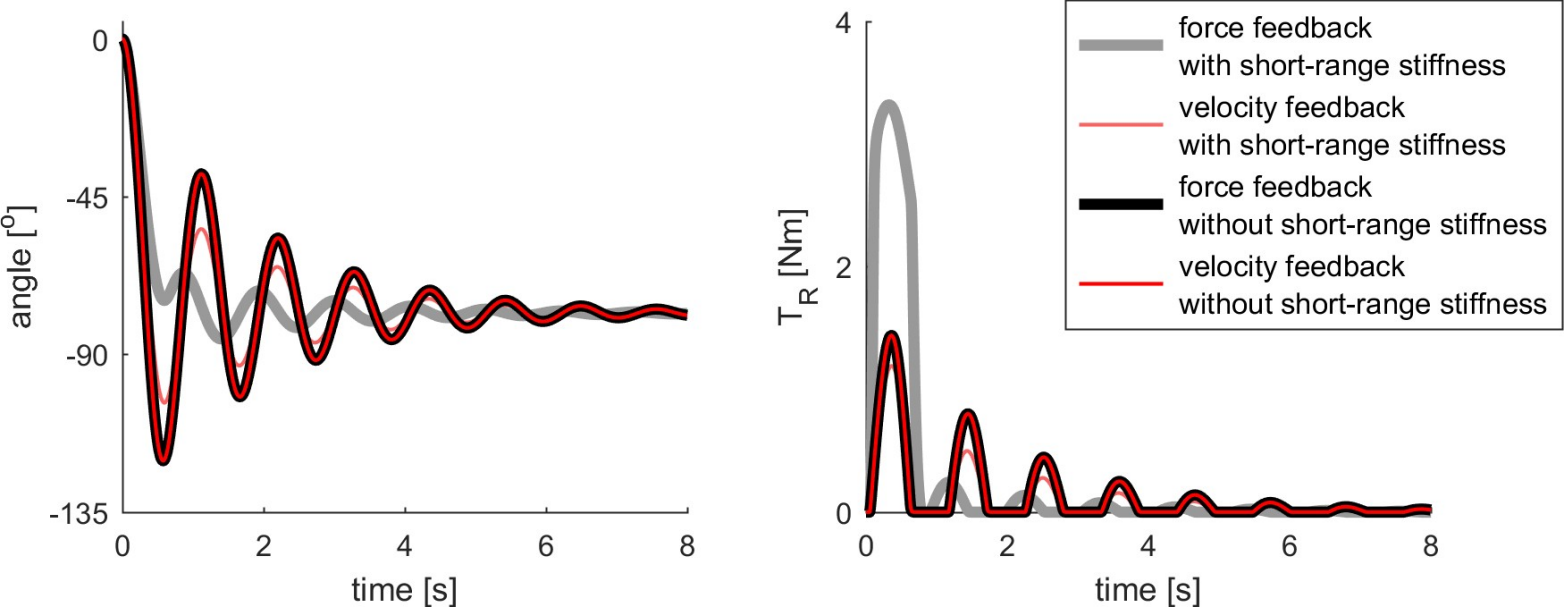
Comparison of force and velocity feedback in the absence of short-range stiffness. Force and velocity reflex gains were chosen such that both models resulted in identical kinematics in the absence of short-range stiffness. All other parameters were as reported in Fig 4 (table). Results from the model with short-range stiffness were added for reference. **(Left)** Kinematic trajectories. **(Right)** Reflex torques.

The differences in force-based and velocity-based reflexes in our model are due to the presence of history-dependent short-range stiffness in our model. The time course of the force and velocity of the simulated muscle differs the most during history-dependent conditions [13]. Therefore force-based and velocity-based reflex feedback differed during the first swing excursion. However, force-based and velocity-based reflex feedback could both explain pendulum test kinematics in subsequent oscillations, and produced identical pendulum test kinematics in the absence of short-range stiffness (Fig 7).

Although hamstrings activity has been observed during extension of the knee in severe spasticity [3], we did not model the action of antagonistic muscles. Accounting for antagonistic muscles might allow us to better fit both peak flexion and extension angles, whereas in the current model we were unable to exactly fit the peak extension angle after the first oscillation in individuals with mild and severe spasticity (Fig 6C and 6D).

In agreement with experimental studies, our model predicted a contribution of baseline tone to pendulum test kinematics in healthy individuals, and a major role of hyperreflexia during the first swing in individuals with spastic CP. Fee and Miller [26] provide evidence in support of non-zero muscle tone in healthy children, as pendulum test lower limb excursion amplitudes are smaller in awake versus anesthetized conditions. While it is difficult to experimentally confirm low levels of activation EMG recordings, we obtained a better fit between simulated and measured kinematic trajectories for the healthy individual when assuming baseline tone levels corresponding to 1 to 2% of maximal muscle activation. In addition, the pendulum test has been shown to be sensitive to reduced muscle tone (hypotonia) in individuals with Down syndrome [27]. In agreement with the EMG recordings reported by Fowler at al. [3], our model predicted reflex activity during the first swing excursion when fitting experimental kinematics of individuals with spastic CP (Fig 6B and 6D). Although in the absence of reflexes, short-range stiffness and increased muscle tone can contribute to each of the key kinematic changes in the pendulum test in spasticity (Fig 3), reflexes were necessary to fit experimental data, supporting the important role of hyperreflexia in spasticity.

In contrast to prior models, we explained the whole range of pendulum test kinematics observed across healthy and CP individuals with different levels of spasticity by varying only two parameters, i.e. constant baseline torque and force-based reflex feedback gain (see table in Fig 6), which correspond directly to increased muscle tone and reflex hyperexcitability in CP. Because we explicitly modeled short-range stiffness, our model required fewer parameters than previous models to describe pendulum test kinematics, and these parameters were directly related to underlying physiological mechanisms. Although the contribution of short-range stiffness to pendulum test kinematics was suggested previously, it was not explicitly simulated. Lin and Rymer used a non-constant joint stiffness throughout the pendulum motion, motivated by muscle short-range stiffness, i.e. thixotropy [4]. He et al. used an activation-dependent reflex gain to account for the higher stiffness of active versus passive muscle [5]. Similarly, other models of the pendulum test in spasticity required changes in model parameters without an underlying mechanistic basis to reproduce pendulum kinematics in spasticity (Table 1). Our simulation of the pendulum test is more parsimonious and describes the physiological effects of increased muscle tone and hyperreflexia. Using a simple torque driven model, in line with most previously proposed models, allowed us to systematically investigate the contribution of all model components to pendulum kinematics. The addition of more details to our model might improve its ability to reproduce fine details of pendulum kinematics but would not qualitatively change our findings. For example, our torque-driven model did not account for the force-length-velocity relation of muscle that induce changes in muscle force upon stretch. Damping in our model can be considered a very simple approximation of the force-velocity properties of muscle. Using a more accurate model describing damping of non-contractile tissues and the force-length-velocity properties separately might alter fine details of pendulum test kinematics but will have little effect on the key kinematic changes since the response to stretch due to short-range stiffness is many times larger. Similarly, altered muscle-tendon properties in CP individuals could contribute to changes in pendulum test kinematics, but these contributions may not be significant, as Fee and Miller [26] demonstrated nearly normal kinematic trajectories in children with CP under anesthesia and given muscle relaxants. In some cases, fewer oscillations were observed under anesthesia, but the decreased first swing excursion and non-vertical resting angle were not observed, suggesting that alterations in passive tissues in CP cannot explain most key feature of the pendulum test that are associated with spasticity.

Our study suggests that using the pendulum test with a physiologically-plausible simulation could help to identify differential causes of spasticity in individuals with CP, as well as help to understand causes of spasticity in other neurological disorders. Our model could be used to test the differential contribution of muscle tone and hyperreflexia in CP individuals. Parameters estimated by fitting modeled and measured kinematics could reveal elevated muscle tone and reflexes at preclinical levels of spasticity (MAS = 1). Additionally, the model could be used to identify targets for rehabilitation or pharmacological treatments that reduce spasticity. The pendulum test has been used to assess spasticity in other patient groups such as stroke [28], spinal cord injury [29], and multiple sclerosis [30]. Our model could be used to investigate whether the same or different phenomena are involved in different patient groups and when spasticity is caused by a motor cortex lesion such as in CP and stroke versus peripheral nerve damage such as in spinal cord injury and multiple sclerosis. Furthermore, the pendulum test has been used to assess rigidity in patients with Parkinson’s disease [28], where accounting for short-range stiffness might also improve existing models of the pendulum test for rigidity [31].

## Material and methods

### Dynamic model

Our model consists of a planar lower leg segment actuated with a passive torque representing non-contractile musculotendon properties, T_pas_, and an active torque representing muscle contractile behavior, T_act_. Knee joint motion is described by

$$J\ddot{\theta }+T_{pas}+T_{act}=m∙g∙l_{c}∙\cos\theta ,$$ (1)

where θ is the knee joint angle (θ = 0 corresponds to full extension and negative values correspond to knee flexion), J is the moment of inertia of the lower leg with respect to the knee, m is the mass of the lower leg, g is gravitational acceleration, and l_c_ is the distance between the knee joint center and the center of mass of the lower leg (Fig 1B). Inertial parameters are taken from OpenSim’s gait2392 model assuming that the ankle is fixed in the anatomical position [14,32]. Inertial parameters are scaled based on the relative length of an individual with respect to the model’s length, s:

$$l_{c}=s∙l_{c0},m=s^{2}∙m_{0},J=s^{4}∙J_{0},$$ (2)

where subscript 0 indicates generic model parameters: l_c0_ = 0.256m, m = 4.96kg, J_0_ = 0.446kgm^2^.

The passive torque consists of a damper T_d_, in parallel with a coordinate limit torque T_lim_, representing the increased joint stiffness towards the limits of the range of motion. Damping torque is

$$T_{d}=B∙\dot{\theta },$$ (3)

where B is the damping constant. Coordinate limit force is

$$T_{\lim}=-\exp\left(k_{\lim}(\theta -\theta_{\mathrm{flex}})\right)+\exp(k_{lim}(-\theta -\theta_{\mathrm{ext}})),$$ (4)

where k_lim_ = 3 is a constant and [θ_flex_ θ_ext_] = [-110^o^ 0^o^] defines the interval of joint angles for which passive stiffness is small, outside this interval the torque due to passive stiffness increases rapidly (Fig 1C).

The active torque consists of a baseline torque to represent muscle tone, T_b_; a short-range stiffness torque, T_SRS_; and a delayed sensory feedback torque to simulate reflex activity, T_R_:

$$T_{act}=T_{b}+T_{SRS}+T_{R}.$$ (5)

The baseline torque represents muscle tone and is constant over the time course of a simulation.

To model the torque arising from muscle short-range stiffness, we adapted the dynamic model of the short-range stiffness component of muscle force that we recently proposed [12] to represent short-range stiffness of the knee extensors at the joint level. We modeled short-range stiffness to be proportional to the isometric torque prior to the stretch (the baseline torque in our model) and included the transient behavior of short-range stiffness by accounting for the critical stretch, i.e. the stretch at which the slope of the force build-up sharply decreases. T_SRS_ increases with knee joint angle change in the direction opposing knee joint motion up to a critical change in knee joint angle, Δθ_c_, after which T_SRS_ is constant for the remainder of the stretch (similar to observations in isolated muscle fibers [10]):

$$T_{SRS}=\left\{ \begin{array}{r} {-k}_{SRS}\;T_{b}\;\Delta \theta ,\;|\Delta \theta |<\Delta \theta_{c}\\ {-k}_{SRS}\;T_{b}\;sign(\Delta \theta )\Delta \theta_{c},\;|\Delta \theta |>\Delta \theta_{c} \end{array}\right.,$$ (6)

where Δθ is the change in joint torque angle from the isometric position, sign(Δθ) is the sign of Δθ, and k_SRS_ is the short-range stiffness constant (Fig 1D). A change in the sign of the angular velocity of the knee indicates the end of the stretch and the start of the exponential decay of the short-range stiffness torque:

$$\frac{dT_{SRS}}{dt}=\frac{-T_{SRS}}{\tau_{SRS}},$$ (7)

where τ_SRS_ = 50ms is the time constant of the exponential decay. Since movement of the muscle prior to the stretch reduces short-range stiffness [20], we assumed that short-range stiffness only contributed to the first swing excursion. We chose Δθ_c_ = 1.5^o^ since a change in knee joint angle of 1.5^o^ corresponds to critical stretch of the rectus femoris muscle based on critical sarcomere stretch reported by Getz et al. [10]. The nominal value of the short-range stiffness constant k_SRS_ = 1Nm/Δθ_c_ corresponds to a short-range stiffness torque that is about twice the baseline torque, which is in accordance with measured increases in muscle fiber force due to short-range stiffness [10,20].

Reflex muscle activity of the knee extensor muscles, e_R_, was modeled as delayed feedback from either joint position and velocity (velocity-based reflex model) or torque and derivative of torque (force-based reflex model):

$$e_{R}=\max[{0,k}_{x}x(t-\gamma )+k_{dx}\dot{x}(t-\gamma )],$$ (8)

where x is either θ or T_SRS_ + T_d,_ k_x_ and k_dx_ are feedback gains and *γ* = 50ms is the time delay of the reflex loop. The reflex muscle activity results in a reflex torque T_R_:

$$\frac{dT_{R}}{dt}=\frac{e_{R}-T_{R}}{\tau_{a}},$$ (9)

where τ_a_ = 10ms is the time constant of muscle activation and contraction dynamics. Due to fusimotor control, stretch reflexes might be insensitive to torques resulting from baseline muscle tone (T_b_) or reflex muscle activity (T_R_). Therefore, only the torque resulting from short-range stiffness (T_SRS_) and damping (T_d_) was included in the reflex model. The underlying assumption here is that the force-velocity properties of muscles, and not connective tissues, are the main contributors to damping.

### Forward simulations

We performed forward dynamics simulations to assess whether our model could reproduce the key kinematic features of the pendulum test in healthy and spastic individuals as described by Fowler et al. [3]. First, we created a control simulation that reproduced healthy pendulum kinematics using only passive elements (damper and coordinate limit torque) in our model. To test our hypothesis that leg excursion during the pendulum test in spastic individuals could be explained by increased baseline muscle activity and sensory feedback gains when muscle short-range stiffness is accounted for, we increased the baseline torque and feedback gains while holding all other model parameters constant. Based on this nominal simulation, we explored the contribution of baseline torque, short-range stiffness and feedback gains to the amplitude of the first swing excursion, the number of oscillations, and the resting angle.

To gain insight in the interaction between model parameters, we performed three sets of forward simulations in which we systematically varied model parameters. First, we varied baseline torque T_b_ and short-range stiffness constant k_SRS_ in the absence of reflex activity. Second, we varied reflex gains in the length- and force-based reflex models when either accounting for or omitting muscle short-range stiffness. Third, we simultaneously varied baseline torque T_b_ and torque feedback gain k_T_. We performed simulations for baseline torques ranging from 0 to 2.5Nm with intervals of 0.5Nm and torque feedback gains ranging from 0 to 2.5 with intervals of 0.25, resulting in a total of 121 simulation.

Finally, we assessed whether our model could reproduce kinematic trajectories of the pendulum test in a healthy and three CP individuals with different levels of spasticity as reported by Fowler et al. [3]. To this aim, we manually tuned model parameters describing the size of the subject, damping, baseline torque, and reflex gains while keeping all other model parameters constant. We tuned model parameters such that the first swing excursion, the resting angle and the frequency corresponded to the experimental data. In our model, frequency was determined by subject’s size. Subjects in Fowler’s study were as young as 7 years, hence we allowed a large range of sizes.

The code to run these simulations is available on simtk.org/projects/pendulumtest.
